# Interactions of PatA with the Divisome during Heterocyst Differentiation in *Anabaena*

**DOI:** 10.1128/mSphere.00188-20

**Published:** 2020-05-20

**Authors:** Ana Valladares, Cristina Velázquez-Suárez, Antonia Herrero

**Affiliations:** a Instituto de Bioquímica Vegetal y Fotosíntesis, CSIC, Seville, Spain; b Universidad de Sevilla, Seville, Spain; University of Wyoming

**Keywords:** *Cyanobacteria*, bacterial multicellularity, cell differentiation, cell division

## Abstract

The *Anabaena* organismic unit is a filament of communicating cells. Under conditions of nitrogen scarcity, some cells along the filament differentiate into heterocysts, which are specialized in the fixation of atmospheric N_2_ and provide the vegetative cells with N_2_ fixation products. At a certain stage, the differentiation process becomes irreversible, so that even when nitrogen is replenished, no return to the vegetative cell state takes place, possibly as a consequence of loss of cell division capacity. Upon N-stepdown, midcell FtsZ-rings were detected in vegetative cells, but not in differentiating cells, and this was also the case for ZipN, an essential protein that participates in FtsZ tethering to the cytoplasmic membrane and divisome organization. Later, expression of *ftsZ* was arrested in mature heterocysts. PatA is a protein required for the differentiation of intercalary heterocysts in *Anabaena*. The expression level of the *patA* gene was increased in differentiating cells, and a mutant strain lacking PatA exhibited enhanced FtsZ-rings. PatA was capable of direct interactions with ZipN and SepF, another essential component of the *Anabaena* Z-ring. Thus, PatA appears to promote inhibition of cell division in the differentiating cells, allowing progress of the differentiation process. PatA, which in mature heterocysts was detected at the cell poles, could interact also with SepJ, a protein involved in production of the septal junctions that provide cell-cell adhesion and intercellular communication in the filament, hinting at a further role of PatA in the formation or stability of the intercellular structures that are at the basis of the multicellular character of *Anabaena*.

**IMPORTANCE** *Anabaena* is a cyanobacterial model that represents an ancient and simple form of biological multicellularity. The *Anabaena* organism is a filament of cohesive and communicating cells that can include cells specialized in different tasks. Thus, under conditions of nitrogen scarcity, certain cells of the filament differentiate into heterocysts, which fix atmospheric nitrogen and provide organic nitrogen to the rest of cells, which, in turn, provide heterocysts with organic carbon. Heterocyst differentiation involves extensive morphological, biochemical, and genetic changes, becoming irreversible at a certain stage. We studied the regulation during heterocyst differentiation of several essential components of the *Anabaena* cell division machinery and found that protein PatA, which is required for differentiation and is induced in differentiating cells, interacts with essential cell division factors and destabilizes the cell division complex. This suggests a mechanism for establishment of commitment to differentiation by inhibition of cell division.

## Abstract (precis)

*Anabaena* is a cyanobacterial model that represents an ancient and simple form of biological multicellularity. The *Anabaena* organism is a filament of cohesive and communicating cells that can include cells specialized in different tasks. Thus, under conditions of nitrogen scarcity, certain cells of the filament differentiate into heterocysts, which fix atmospheric nitrogen and provide organic nitrogen to the rest of cells, which, in turn, provide heterocysts with organic carbon. Heterocyst differentiation involves extensive morphological, biochemical, and genetic changes, becoming irreversible at a certain stage. We studied the regulation during heterocyst differentiation of several essential components of the *Anabaena* cell division machinery and found that protein PatA, which is required for differentiation and is induced in differentiating cells, interacts with essential cell division factors and destabilizes the cell division complex. This suggests a mechanism for establishment of commitment to differentiation by inhibition of cell division.

## INTRODUCTION

*Anabaena* sp. strain PCC 7120 (here referred to as *Anabaena*) is a model of filamentous cyanobacteria able to undertake a developmental program to form a multicellular diazotrophic bacterium. In response to nitrogen shortage, some cells of the filament differentiate into cells specialized in the fixation of atmospheric nitrogen (N_2_), called heterocysts, which are distributed in a semiregular pattern of two heterocysts separated by an average of ca. 10 vegetative cells along the filament. Thus, the diazotrophic filament is composed of two cell types that exchange nutrients and regulatory molecules: the vegetative cells, which fix CO_2_ and transfer C-rich compounds (sugars) to heterocysts, and the heterocysts, which fix N_2_ and donate N-rich compounds (amino acids) to the vegetative cells (1). Additionally, at least one morphogen, PatS, is also transferred from the differentiating cells to inhibit differentiation of its neighbors, thus contributing to the setting of the heterocyst distribution pattern (2, 3). Several septal proteins, including SepJ, FraC, and FraD (4), have been implicated in the formation of the septal junctions that bridge the cytoplasms of neighboring cells and provide cohesion and intercellular communication along the filament.

Vegetative cells and heterocysts differ in multiple morphological and physiological features as the result of largely different gene expression programs that are established in each cell type during the differentiation process, including multiple events of transcriptional regulation (the process that has been most extensively studied) but also events of posttranslational regulation, which are currently being dissected (5). Two principal transcriptional regulators are required for differentiation: the cAMP receptor protein (CRP)-family type NtcA global factor, which mediates regulatory responses to changes in the C-to-N balance of the cells (6), and HetR, which mediates gene activation in the differentiating cells (7).

Heterocysts are terminally differentiated cells, which implies that beyond a certain point in the differentiation process no reversion to the vegetative cell entity takes place, even when the cue that triggers differentiation, i.e., nitrogen deficiency, is abrogated (8, 9). In the mature diazotrophic filament, old heterocysts are thought to undergo senescence and finally go dead. Irreversibility of heterocyst differentiation can be related to loss of cell division capacity, which is assumed to take place roughly coincidently with the commitment to differentiation. FtsZ has been reported to occur at lower levels in mature heterocysts than in vegetative cells (10, 11). However, the mechanism responsible for an asymmetric distribution of FtsZ in the diazotrophic filament is not known. Other possible regulatory events that may result in inhibition of cell division during heterocyst differentiation are also unknown.

Several genes that are required for heterocyst differentiation have been related to the step of commitment. This is the case with *hetC*, encoding an ABC-type exporter (12) localized in the heterocyst poles that is involved in transferring the PatS morphogen to neighboring cells (13), and with *hetP* and some *hetP* homologs (14). Judging from the morphological effects of their inactivation or overexpression, two other factors that regulate heterocyst differentiation, HetF and PatA, might affect cell division. Thus, inactivation of the *hetF* gene leads to the presence of enlarged and elongated cells, and ectopic overexpression of *hetF* leads to the presence of cells smaller than those of the wild type (WT) throughout the filament (15). The PatA protein of *Anabaena* bears a CheY-like phosphoacceptor domain in its C terminus as well as a so-called PATAN domain of undetermined function (16). Inactivation of *patA* results in a phenotype of a low frequency of heterocysts that, moreover, are mainly found at the filament ends and in consequent poor growth under diazotrophic conditions (17), whereas *patA* overexpression increases the frequency of heterocysts (18).

To gain insight into the mechanism of inhibition of cell division during heterocyst differentiation, we have monitored the expression and localization of initial cell division factors and PatA along the filament during the differentiation process. We have also studied the effects of *patA* deletion on the localization of the FtsZ-ring, as well as possible interactions between PatA and proteins involved in cell division or intercellular communication.

## RESULTS

### Expression and localization of FtsZ during heterocyst differentiation.

**FIG 1:**
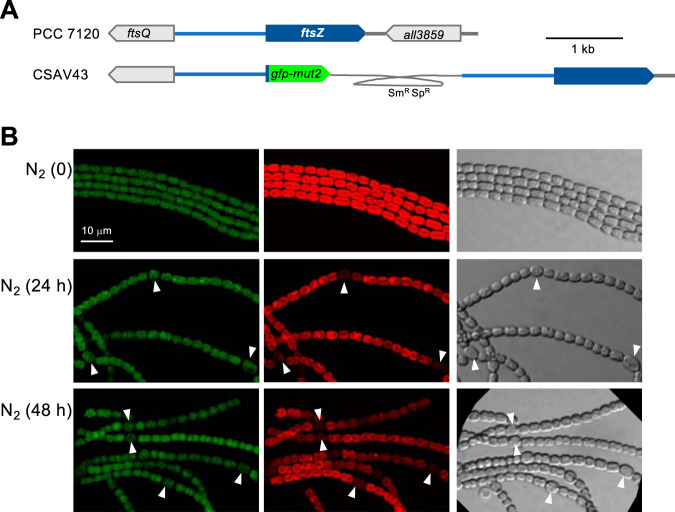
Expression of the *Anabaena ftsZ* gene promoter. (A) Schematic of the genome structure in the *ftsZ* region of strain CSAV43 (P*_ftsZ_*-*gfp*) in comparison to PCC 7120 (WT). The gray trace represents an inserted plasmid encoding resistance to Sm and Sp (see Materials and Methods for details). (B) Filaments of strain CSAV43 grown in BG11 medium were transferred (at a cell density of 2 μg Chl/ml) to BG11_0_ (no combined nitrogen) medium (N_2_) and incubated under culture conditions. After the times indicated, filaments were observed by confocal microscopy and photographed. Time 0 denotes the start of incubation in BG11_0_. GFP fluorescence (green), cyanobacterial autofluorescence (red), and bright-field images are shown. Arrowheads point to heterocysts. The magnification is the same for all micrographs.

To study the expression from the *ftsZ* gene promoter, we generated strain CSAV43, bearing a copy of the *ftsZ* promoter fused to *gfp-mut2* in the native gene locus and keeping an intact copy of the gene expressed from the native promoter (Fig. 1A). The spatiotemporal pattern of expression of P*_ftsZ_*-*gfp* was monitored in cells of strain CSAV43 grown with nitrate and transferred to medium lacking combined nitrogen, which triggers heterocyst differentiation (Fig. 1B). At the onset of N deprivation, filaments showed similar levels of green fluorescent protein (GFP) fluorescence in all the cells. Then, certain cells along the filament differentiated to heterocysts, so that after 24 h many regularly spaced mature heterocysts, recognized by loss of red fluorescence (due to dismantlement of photosystem II) and by cell morphology (including increased cell size and the presence of refringent polar granules made of cyanophycin, a product of N_2_ fixation), could be detected. At that time, most heterocysts exhibited GFP fluorescence similar to that in neighboring vegetative cells (the mean level of fluorescence intensity in heterocysts was ca. 1.1 times that in vegetative cells, as counted over 40 heterocysts and 341 vegetative cells). After 48 h, GFP fluorescence in heterocysts became noticeably lower that in vegetative cells (the mean level of fluorescence intensity in heterocysts was ca. 0.7 times that in vegetative cells, as counted over 40 heterocysts and 255 vegetative cells). Thus, the *ftsZ* promoter was downregulated in heterocysts well after the differentiation process has been completed.

**FIG 2:**
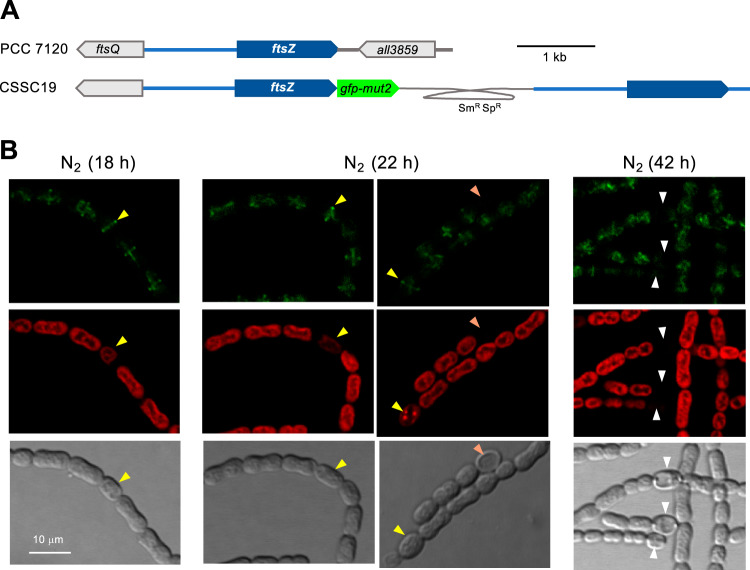
Localization of FtsZ during heterocyst differentiation in *Anabaena*. (A) Schematic of the genome structure of strain CSSC19 (*ftsZ*-*gfp-mut2*) (19) in comparison to PCC 7120 (WT). The gray trace represents an inserted plasmid encoding resistance to Sm and Sp. (B) Filaments of strain CSSC19 grown in BG11 medium were transferred (at a cell density of 2 μg Chl/ml) to BG11_0_ medium and incubated under culture conditions. After the times indicated, filaments were observed by confocal microscopy and photographed. GFP fluorescence (green), cyanobacterial autofluorescence (red), and bright-field images are shown. Arrowheads point to proheterocysts (yellow), immature heterocysts (orange), and mature heterocysts, exhibiting polar refringent granules (white). The magnification is the same for all micrographs.

We have also studied the localization of FtsZ upon N-stepdown in *Anabaena* strain CSSC19, which expresses a *ftsZ-gfp-mut2* gene fusion placed at the *ftsZ* locus and which keeps an intact copy of *ftsZ* also expressed from the native promoter (19) (Fig. 2A). As shown in Fig. 2B, at 18 h after nitrate removal, GFP fluorescence could be detected in midcell rings in all the cells of the filament, including differentiating cells (the mean level of fluorescence intensity in proheterocysts was ca. 1.2 times that in vegetative cells, as counted over 9 proheterocysts and 126 vegetative cells). By 22 h, proheterocysts still exhibiting high GFP fluorescence, together with immature heterocysts (recognized by a thickened cell envelope) showing low GFP fluorescence, could be detected (the mean levels of fluorescence intensity in proheterocysts and immature heterocysts were ca. 0.9 and 0.2 times that in vegetative cells, respectively, as counted over 23 proheterocysts, 20 immature heterocysts, and 238 vegetative cells). After 42 h, GFP fluorescence was high in vegetative cells and low in mature heterocysts (which exhibited conspicuous polar granules) (the mean level of fluorescence intensity in heterocysts was ca. 0.2 times that in vegetative cells, as counted over 11 heterocysts and 255 vegetative cells). Thus, Z-rings appear to be lost during the process of heterocyst differentiation.

### Downregulation of ZipN during heterocyst differentiation.

**FIG 3:**
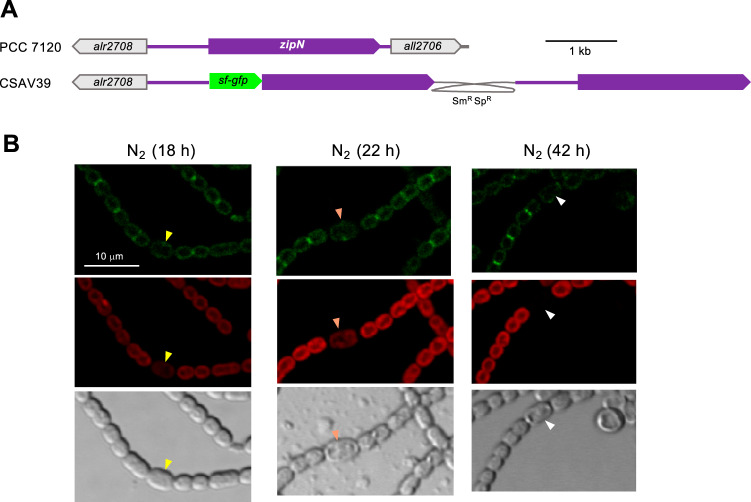
Localization of ZipN during heterocyst differentiation in *Anabaena*. (A) Schematic of the genome structure of strain CSAV39 (*sf-gfp*-*zipN*) in comparison to PCC 7120 (WT). (B) Filaments of strain CSAV39 were treated as described in the legend for Fig. 2, observed by confocal microscopy, and photographed. Arrowheads point to proheterocysts (yellow), immature heterocysts (orange), and mature heterocysts that exhibit polar refringent granules (white). The magnification is the same for all micrographs.

ZipN is an FtsZ-interacting protein that contributes to FtsZ tethering to the cytoplasmic membrane and is an essential part of the Z-ring in *Anabaena* (20). To study the localization of ZipN during heterocyst differentiation, we generated *Anabaena* strain CSAV39, which bears a version of the *zipN* gene 5′ fused to the *sf-gfp* gene (encoding superfolder GFP) in the *zipN* locus (Fig. 3A). Upon nitrate withdrawal, GFP fluorescence localized in midcell rings and in intercellular regions could be detected in vegetative cells but not in either proheterocysts (see 18-h data in Fig. 3B) or heterocysts (see 22-h and 42-h data). This shows that the ZipN protein levels are negatively regulated during heterocyst differentiation.

### Morphological features of a *patA* mutant.

**FIG 4:**
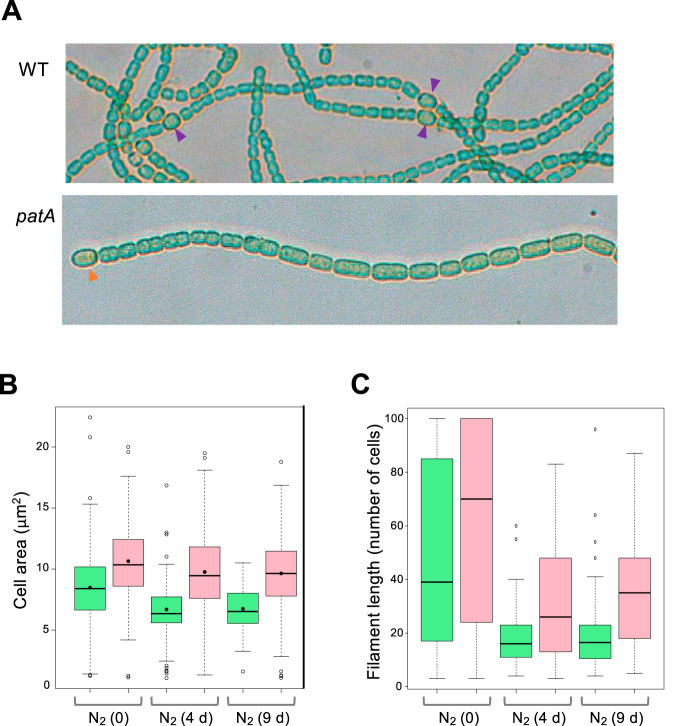
Cell and filament size in a *patA* mutant. Strains PCC 7120 (WT) and UHM101 (*patA* mutant) grown in BG11 medium (NO_3_^−^) were transferred to BG11_0_ medium (N_2_) and incubated under culture conditions. At time zero and after 4 and 9 days on incubation in BG11_0_ medium, filaments were observed with a bright-field microscope and photographed. (A) Filaments after 4 days of incubation in BG11_0_. Purple arrowheads indicate intercalary heterocysts and orange arrowheads terminal heterocysts. (B) Photographs were used for determination of cell area (vegetative cell area in the case of N_2_ cultures) as described in Materials and Methods. A total of 200 to 250 cells of each strain were measured under each condition. A box plot representation of the data is shown. Mean values are represented by black dots. (C) Filament length was counted as the number of cells per filament over 50 to 60 filaments for each strain and condition. Filaments with more than 100 cells were counted as representing 100 cells. WT, green; *patA* mutant, pink.

Strain UHM101, an *Anabaena* mutant in which the coding region of the *patA* gene was deleted, forms predominantly terminal heterocysts (21) (see Fig. 4A). Here, we studied cell and filament size in strain UHM101 in comparison to wild-type *Anabaena*. Cell area was significantly larger in the *patA* mutant than in the wild type, both after incubation with nitrate (mean cell area, 8.47 μm^2^ for the wild type [WT] and 10.65 μm^2^ for the *patA* mutant) and, in particular, after incubation in the absence of combined nitrogen (mean vegetative cell area after 4 days under diazotrophic conditions, 6.69 μm^2^ WT and 9.76 μm^2^ *patA* mutant; mean vegetative cell area after 9 days, 6.74 μm^2^ WT and 9.64 μm^2^ *patA* mutant) (Fig. 4A and B). In addition, filaments were considerably longer in the mutant than in the wild type (Fig. 4C).

### Expression and localization of PatA.

**FIG 5:**
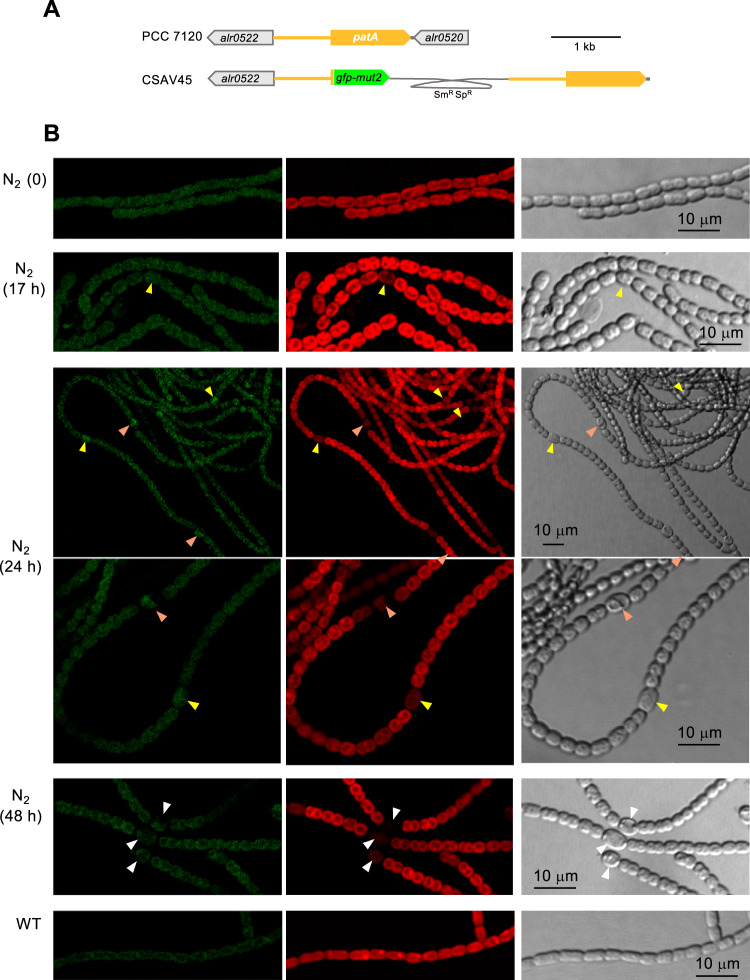
Expression of the *Anabaena patA* gene promoter. (A) Schematic of the genome structure of strain CSAV45 (P*_patA_*-*gfp*) in comparison to PCC 7120 (WT). The gray trace represents an inserted plasmid encoding resistance to Sm and Sp (see Materials and Methods for details). (B) Filaments of strain CSAV45 grown in BG11 medium were transferred (at a cell density of 2 μg Chl/ml) to BG11_0_ medium and incubated under culture conditions. After the times indicated, filaments were observed by confocal microscopy and photographed. Time 0 denotes the start of incubation in BG11_0_. GFP fluorescence (green), cyanobacterial autofluorescence (red), and bright-field images are shown. Arrowheads point to proheterocysts (yellow), immature heterocysts (orange), and mature heterocysts that exhibit polar refringent granules (white).

The expression of the *patA* gene has been described as increasing upon N-stepdown, but always taking place at low levels (17, 22). To study the spatiotemporal pattern of *patA* expression during differentiation, we generated *Anabaena* strain CSAV45, which expresses a fusion of the *patA* gene promoter to *gfp-mut2* in the *patA* locus (Fig. 5A). Fluorescence was monitored in filaments of strain CSAV45 grown with nitrate and incubated during different times after removal of nitrate (Fig. 5B). Only low levels of GFP fluorescence were detected in strain CSAV45, although even at time zero (nitrate growth), the level of fluorescence was noticeably higher than in wild-type *Anabaena* (not bearing the *gfp* gene). Upon N-stepdown, GFP fluorescence increase was detected after 24 h, in cells that still resemble proheterocysts and immature heterocysts. By 48 h, GFP fluorescence in mature heterocysts had decreased to basal levels. Thus, activation of *patA* expression during heterocyst differentiation seems to be transitory. (Given the low expression level, we cannot rule out the possibility that the *patA* gene was activated earlier than the time at which accumulation of GFP was detected.)

**FIG 6:**
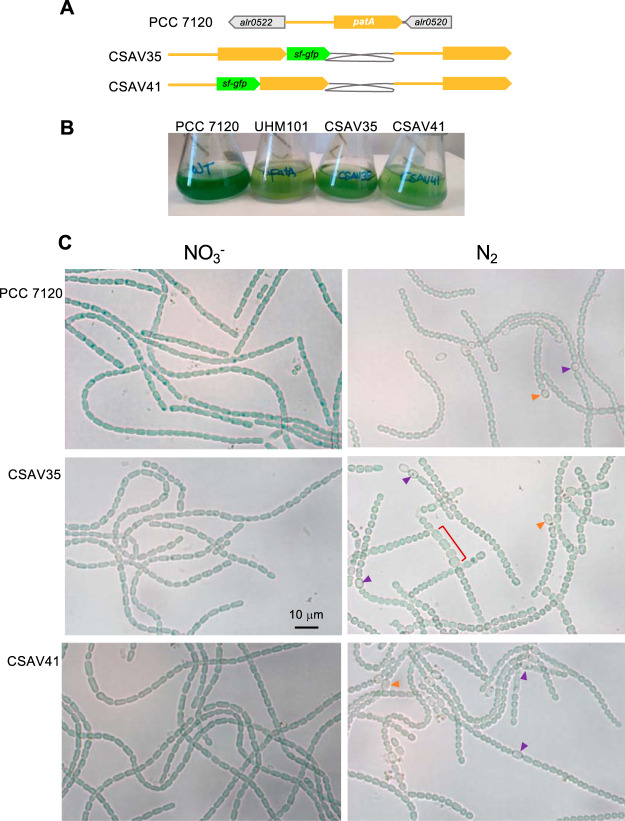
Growth and morphology of strains expressing GFP fusions to PatA. (A) Schematic of the genome structure of strain CSAV35 (*patA*-*sf-gfp*) and strain CSAV41 (*sf-gfp-patA*) in comparison to PCC 7120 (WT). The gray trace represents an inserted plasmid encoding resistance to Sm and Sp (see Materials and Methods for details). (B) Cultures in BG11 medium (containing NO_3_^−^) were used to inoculate flasks with BG11_0_ medium (at a cell density equivalent to 1 μg Chl/ml), which were incubated under culture conditions and photographed after 4 days. Strain UHM101 is a *patA* mutant (see the text). (C) Filaments from flasks inoculated with 0.5 μg Chl/ml and photographed after 43 h. Purple arrowheads indicate intercalary heterocysts, orange arrowheads terminal heterocysts, and red brackets stretches of aberrant cells in the filament. The magnification is the same for all micrographs.

To study the localization of PatA, we generated two strains bearing *patA* fusions to *sf-gfp* inserted in the native *patA* locus and maintaining an intact *patA* gene: strain CSAV35 bears a *patA* gene 3′ fused to *sf-gfp* and strain CSAV41 a *patA* gene 5′ fused to *sf-gfp* (Fig. 6A). In media supplemented with nitrate, both strains, CSAV35 and CSAV41, grew well, and their filament morphology was similar to that of the wild type (Fig. 6C). In the absence of combined nitrogen, both strains were able to grow, although growth was somewhat impaired in comparison to that seen with the wild type (Fig. 6B). Strains CSAV35 and CSAV41 were able to differentiate terminal and intercalary heterocysts (Fig. 6C), although, with regard to the wild type, differentiation was somewhat delayed, and the frequency of intercalary heterocysts was lower in CSAV35 than in CSAV41 or the wild type. In both the CSAV35 and CSAV41 mutant strains, occasional aberrant cells could be observed, and those cells were more noticeable under conditions of rapid growth (see Fig. 6C). This suggests some degree of interference with cell division, which might have resulted from the presence of two *patA* copies.

**FIG 7:**
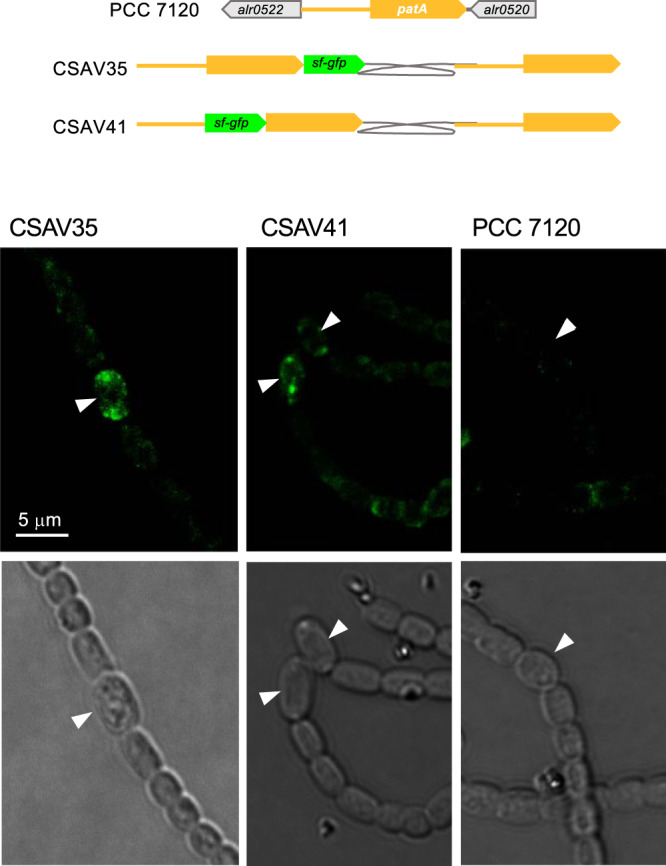
Localization of PatA during heterocyst differentiation. (A) Schematic of the genome structure of strain CSAV35 (*patA*-*sf-gfp*) and strain CSAV41 (*sf-gfp-patA*) in comparison to PCC 7120 (WT). (B) Filaments of strains PCC7120, CSAV35, and CSAV41 grown in BG11 medium were transferred to BG11_0_ medium and incubated under culture conditions. After 22 h, filaments were observed under a fluorescence microscope and photographed. GFP fluorescence (green) and bright-field images are shown. Arrowheads point to heterocysts. The magnification is the same for all micrographs.

GFP fluorescence was monitored in strains CSAV35 and CSAV41. Only occasional weak signals were detected, and, in both strains, they were seen as condensed spots in the polar regions of heterocysts. These signals were absent from strain PCC 7120 (lacking GFP) (Fig. 7).

### Localization of FtsZ in a *patA* mutant background.

**FIG 8:**
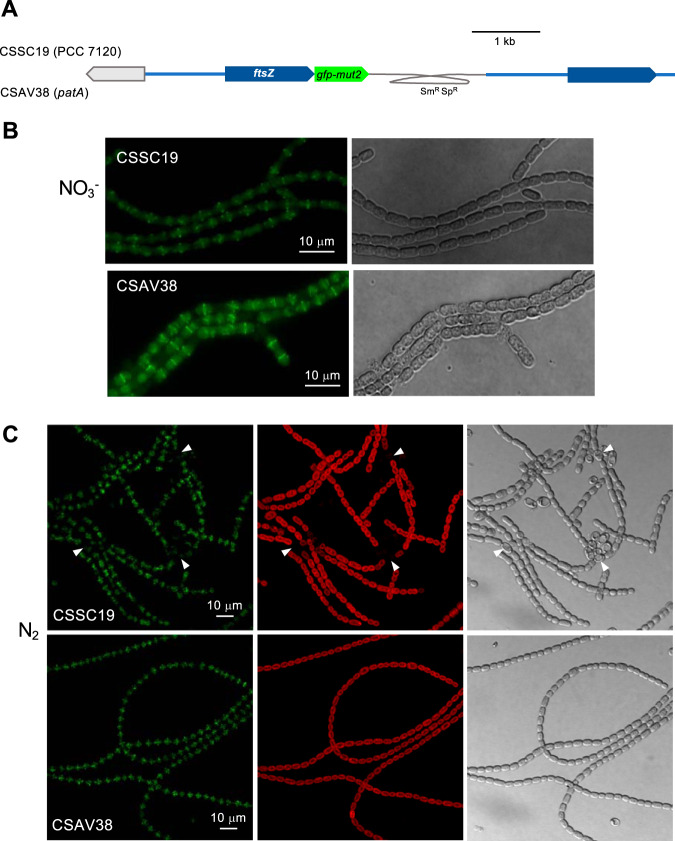
Visualization of FtsZ in a *patA* mutant background. (A) Schematic of the genome structure of strain CSSC19 (*ftsZ*-*gfp*-*mut2* in WT background) (19) and strain CSAV38 (*ftsZ*-*gfp-mut2* in *patA* mutant background) (see Materials and Methods for details). (B) Nitrate-grown filaments were observed under a fluorescence microscope and photographed. (C) Nitrate-grown filaments were incubated for 42 h in BG11_0_ medium and observed with a confocal microscope and photographed. GFP fluorescence (green), cyanobacterial autofluorescence (red), and bright-field images are shown. Arrowheads point to mature heterocysts.

To test whether the mutation of the *patA* gene had any effect on the conformation of FtsZ-rings, we generated strain CSAV38, which bears in UHM101 (*patA* background) the same *ftsZ-gfp-mut2* gene fusion present in strain CSSC19 (wild-type background; see above) (Fig. 8A). In nitrate-grown filaments, both strain CSSC19 and strain CSAV38 showed GFP fluorescence in midcell rings. Notably, the intensity of GFP fluorescence was stronger in CSAV38 than in CSSC19 (the mean midcell fluorescence intensity in CSAV38 was ca. 1.6 times that in CSSC19, as counted over 50 cells of CSAV38 and 59 cells of CSSC19; Student's *t* test, *P* = 10^−17^) (see Fig. 8B for representative images), suggesting that the deletion of *patA* has a positive effect on the formation or stability of the Z-ring. Upon N-stepdown, strain CSAV38, similarly to its parental UHM101 strain, produced mostly terminal heterocysts. After incubation in the absence of combined nitrogen, the levels of fluorescence intensity in vegetative cells of CSSC19 and CSAV38 were similar (the mean midcell fluorescence intensity in CSAV38 was ca. 0.9 times that in CSSC19, as counted over 51 cells of CSAV38 and 55 cells of CSSC19; Student's *t* test, *P* = 0.09). However, a noticeable difference between the two strains was that CSAV38 did not exhibit intercalary cells devoid of GFP fluorescence, as observed in CSSC19 (Fig. 8C; see also Fig. 2).

### PatA interactions with *Anabaena* cell division factors.

**FIG 9:**
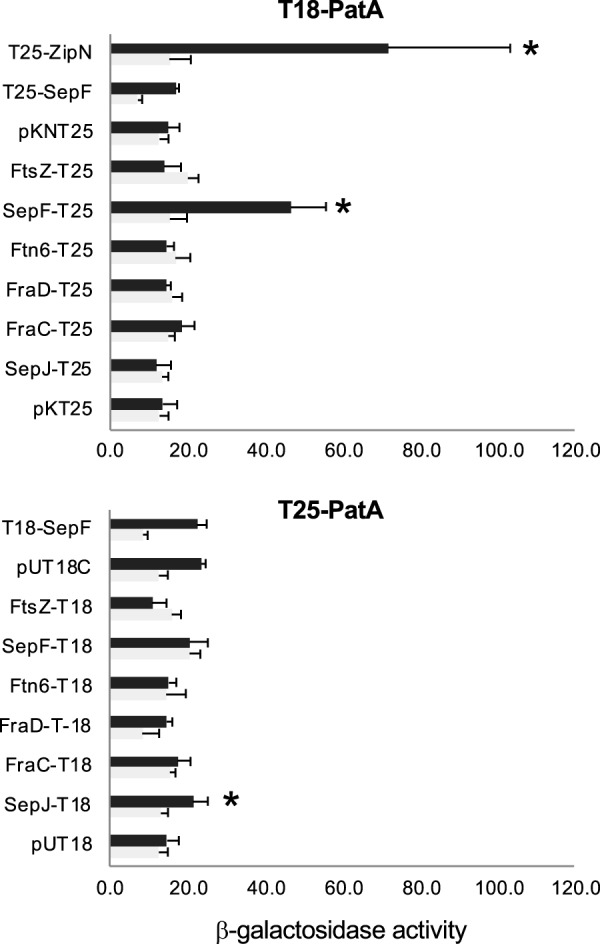
BACTH assays of PatA interactions. Interactions of protein pairs produced in E. coli were assayed by measurements of β-galactosidase activity (nmol ONP min^−1 ^mg protein^−1^) in liquid cultures incubated at 30°C. The topology of each fusion is indicated by the order of components (T18-protein and T25-protein denote the corresponding adenylate cyclase domains fused to the N terminus of the test protein, whereas protein-T18 and protein-T25 denote fusions to the C terminus). Data represent means and standard deviations of 2 to 8 determinations of the activity assayed with the indicated protein fused to T25 (or empty vector pKNT25 or pKT25) and T18-PatA (dark bars) or with the indicated protein fused to T25 (or empty vector pKNT25 or pKT25) and pUT18C (clear bars) (upper part) or with the indicated protein fused to T18 (or empty vector pUT18C or pUT18) and T25-PatA (dark bars) or the indicated protein fused to T18 (or empty vector pUT18C or pUT18) and pKT25 (clear bars) (lower part). The significance of differences was assessed by Student’s *t* tests. Asterisks indicate strains expressing a pair of tested proteins that exhibited β-galactosidase activity significantly different (*P* < 0.01) from that seen with all three controls: the strain containing both empty vectors and the two strains expressing each fused protein and containing the complementary empty vector.

Given the apparent effects of *patA* alterations on cell morphology and detection of the cell division ring, we used bacterial adenylate cyclase two-hybrid system (BACTH) analysis to test possible direct interactions of PatA with identified components of the *Anabaena* Z-ring, namely, the proteins FtsZ, ZipN, Ftn6, and SepF. Also, because of the apparent polar localization of PatA in the heterocysts, we tested interactions with *Anabaena* septal junction proteins SepJ, FraC, and FraD. For that, all four possible versions of PatA (T18-PatA, PatA-T18, T25-PatA, and PatA-T25) were generated. A strong interaction of T18-PatA with T25-ZipN was detected (as already published [20], T25-ZipN was the only ZipN fusion that we were able to clone). A clear interaction was also detected involving T18-PatA and SepF-T25. Finally, a weak but significant interaction was detected between T25-PatA and SepJ-T18 (Fig. 9). No significant interaction was detected involving C-terminal fusions to PatA (i.e., neither PatA-T18 nor PatA-T25), suggesting that these fusions could lead to the production of PatA versions that are not active for BACTH analysis.

**FIG 10:**
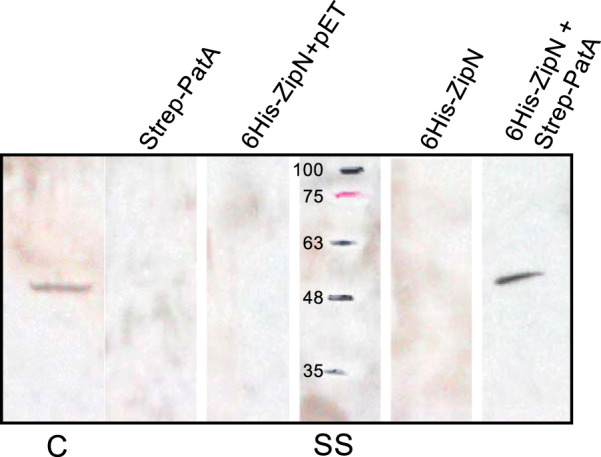
Copurification of PatA and ZipN proteins. Cell-free extracts of E. coli expressing 6His-ZipN or Strep-tag-PatA, or including plasmid vector pET28b, were incubated individually, or in various combinations, at 4°C overnight and were passed through a His-Select column (see Materials and Methods). After loading, the column was washed first with buffer A and then with the buffer supplemented with 300 mM imidazole. Finally, tightly bound proteins were eluted with buffer supplemented with 1 M imidazole. Aliquots of the eluents were subjected to Western blotting with antibodies against Strep-tag. C, purified Strep-tag-PatA (45.257 kDa); SS, size standard (kDa).

The interaction between PatA and ZipN was also assessed by copurification assays. A version of ZipN that included a 6×His tag fused to its N-terminal end and a version with PatA N-terminally fused to a Strep-tag were generated. Cell-free extracts of E. coli expressing each of these proteins alone or mixed together were incubated and then passed through a His-select column, and the presence of Strep-PatA in the eluted fractions was assessed by immunoblotting with antibodies against the Strep-tag and by matrix-assisted laser desorption ionization–time of flight (MALDI-TOF). As shown in Fig. 10, Strep-PatA, as revealed by immunoblotting, was tightly retained in the column only when it had been previously incubated and loaded together with His-ZipN. In the fractions that, according to Western blotting results, contained His-ZipN and Strep-PatA, MALDI-TOF analysis rendered PatA protein sequence coverage of 34% and a Mascot score of 177 (protein scores greater than 93 were considered significant; *P* < 0.05). For ZipN, sequence coverage of 12% and a Mascot score of 102 were obtained. Taken together, the results of the BACTH and copurification analyses indicate that PatA interacts with ZipN.

## DISCUSSION

In the filamentous cyanobacterium *Anabaena*, heterocysts specialized in the fixation of atmospheric nitrogen differentiate in a semiregular spatial pattern under conditions of nitrogen scarcity. Heterocysts are terminally differentiated cells that do not divide or revert to the vegetative-cell state. At an intermediate stage during differentiation, the process becomes irreversible (8, 9). A question arises concerning whether commitment to differentiation is related to loss of cell division capacity and what the mechanism of inhibition of cell division might be during differentiation.

It has been reported that FtsZ is undetectable, by immunoblotting, in cell extracts from mature heterocysts (10). On the other hand, expression of a GFP reporter directed from the *ftsZ* gene promoter placed in a shuttle plasmid was downregulated in heterocysts (23), and a FtsZ-GFP fusion protein expressed from the heterologous *petE* promoter placed in the same shuttle vector was observed in proheterocysts (12 h after N-stepdown) but not in heterocysts (19 or 24 h after N-stepdown) (24). However, these constructs may not reflect the physiological regulation of the *ftsZ* gene and the FtsZ protein because the shuttle vector used to clone the reporters (pRL25c, based on *Nostoc* plasmid pDU1) is maintained in *Anabaena* with variable copy numbers (25) and, moreover, the P*_petE_* promoter itself may be downregulated in heterocysts. Regarding regulation of *zipN*, we are not aware of any previous report showing the expression of the *zipN* gene or the localization of the ZipN protein during heterocyst differentiation. In this work, we have generated strains in which gene and protein reporters are expressed at levels similar to the physiological levels. Thus, in strain CSAV43, the *gfp* fusion to the *ftsZ* promoter preserves all the promoter and translation initiation signals and is placed in the native genomic locus. In CSSC19 and CSAV39, the genes encoding FtsZ and ZipN protein fusions to GFP, respectively, are also placed in their respective native loci. To minimize possible effects of a lack of an intact copy of the proteins on promoter activity or cell physiology, we chose to keep an intact version of the corresponding gene expressed from its native promoter as well.

We have observed that, upon combined nitrogen deprivation, downregulation of the *ftsZ* promoter was a late event, taking place in mature heterocysts (observed 48 h after N-stepdown; Fig. 1), whereas midcell FtsZ-rings were already downregulated in immature heterocysts (Fig. 2). Regarding ZipN, loss of this protein from differentiating cells was already observed at early stages, in proheterocysts (Fig. 3). Because ZipN has an essential role in the stability of the Z-ring (20), these observations are consistent with a sequence of regulatory events in which an initial loss of ZipN during differentiation leads to Z-ring destabilization, before downregulation of the *ftsZ* gene, which in turn would result in a permanent incapacity for cell division in the mature heterocyst.

The protein PatA is required for the differentiation of intercalary heterocysts in *Anabaena*. Because terminal cells in the filament contact other cells at only one pole, it can be considered that heterocyst differentiation at the filament ends, such as in *patA* mutants, might be less sensitive to positional information (e.g., information established by intercellularly transferred inhibitors) that impacts the differentiation of intercalary heterocysts (see reference 21). Thus, differentiation at the filament ends could take place even in the absence of some of the regulatory factors required for intercalary differentiation. The *patA* gene is expressed at undetectable levels in the presence of combined nitrogen, and is induced, although always with a low expression level, upon N-stepdown. Some observations suggest that PatA may have a connection to cell division. In a previous report, expression of *patA* from the *petE* promoter determining high overexpression in vegetative cells (construct carried in a plasmid based on pRL444) resulted in enlarged cells with aberrant morphology in both the presence and absence of combined nitrogen, and a PatA-GFP fusion overexpressed equally was concentrated in midcell ring-like structures in dividing cells (18). Although, as recognized by the authors, these effects appear to have been overexpression artifacts, those results might suggest that the PatA protein has an intrinsic capacity of interaction with some component(s) of the *Anabaena* divisome, thus impacting cell division. Under diazotrophic conditions, we have also observed occasional morphological alterations, including an increase in cell size, in strains that express PatA fusions to GFP at physiological levels together with the native PatA (see Fig. 6C), with the alterations being more pronounced under conditions of rapid growth. These observations are consistent with the idea that PatA interferes with cell division.

Previously, GFP directed from a P_*patA*_-*gfp* reporter fusion, placed in plasmid pRL25c, was detected in heterocysts 24 h after N-stepdown (22). Using *gfp* fusions to the *patA* gene promoter in the native genomic locus, we have detected that GFP is transiently accumulated upon N-stepdown, being detected in proheterocysts and immature heterocysts and fading thereafter (Fig. 5). The detection of activation of P*_patA_* in differentiating cells is consistent with the previous description of upregulated transcription from one transcription start point located at 614/645 nucleotides upstream of *patA* (18) and with regulation by NtcA (22). Both PatA-sfGFP (PatA-superfolder GFP) (in strain CSAV35) and sfGFP-PatA (in strain CSAV41) protein fusions are observed at the polar regions of the heterocysts (Fig. 7). Given the low level of expression of the PatA protein, the concentration at the heterocyst poles could facilitate visualization in this position. However, this might not be the only localization of PatA. Indeed, the previous hints of PatA localization in cell division complexes are extended by our results showing PatA interactions with the essential components of the *Anabaena* divisome ZipN and SepF. Taking all these results together, it can be suggested that during heterocyst differentiation, PatA might first interact with ZipN (and SepF), with the effect of destabilizing the FtsZ-ring, thus mediating inhibition of cell division. This inhibitory effect is supported by our observation that the levels of FtsZ-GFP in midcell Z-rings are higher in the *patA* mutant than in the wild type (Fig. 8). Upon N-stepdown, the absence of PatA in the mutant would favor persistence of divisional complexes in all the cells of the filament, with the effect of impeding the progression of differentiation. A first interaction of PatA with ZipN is consistent with the strong interaction between these proteins observed in BACTH assays. In summary, we propose that at a certain stage during heterocyst differentiation, PatA inhibits the establishment of the cell division complex to allow completion of differentiation. Later, PatA would localize to the cell poles (as seen in Fig. 7), where it could interact with SepJ, consistent with the significant PatA-SepJ interactions detected in BACTH assays. By acting on SepJ, PatA could affect intercellular communication between heterocysts and its neighboring vegetative cells, an issue worth of further investigation.

## MATERIALS AND METHODS

### Strains and growth conditions.

*Anabaena* sp. strain PCC 7120 and strains UHM101, CSSC19, CSAV35, CSAV38, CSAV39, CSAV41, CSAV43, and CSAV45 were grown in BG11 medium (which includes NaNO_3_ as a nitrogen source) (26) containing ferric citrate instead of ferric ammonium citrate, or in BG11_0_ medium, which lacks NaNO_3_, supplemented with 4 mM NH_4_Cl and 8 mM TES-NaOH [*N*-tris(hydroxymethyl)methyl-2-aminoethanesulfonic acid-NaOH] buffer (pH 7.5). For incubation in the absence of combined nitrogen, BG11_0_ medium was used. Cultures were incubated at 30°C in white light (30 μmol photons m^−2^ s^−1^ from LED lamps), in shaken liquid media, or in plates solidified with 1% agar. For strains CSSC19, CSAV35, CSAV38, CSAV39, CSAV41, CSAV43, and CSAV45, the growth medium was supplemented with spectinomycin (Sp) and streptomycin (Sm) at 5 μg ml^−1^ each in solid media and 2 μg ml^−1^ each in liquid media. Chlorophyll content (Chl) of the cultures was determined after extraction with methanol (27). (In *Anabaena*, 1 μg Chl corresponds to ca. 3.3 × 10^6^ cells.)

### Plasmid and strain constructions.

To generate *Anabaena* strains reporting the *ftsZ* (alr3858) or the *patA* (all0521) gene promoter, DNA fragments of 1,037 bp or of 777 bp, encompassing the first six codons and sequences upstream of *ftsZ* or *patA*, respectively, flanked by EcoRV and ClaI sites, were amplified by PCR using *Anabaena* DNA as the template and primers ftsZ-52/ftsZ-53 (all oligodeoxynucleotide primers used are listed in Table 1) (for *ftsZ*) or plasmid pCSAV271 (see below) as the template and primers patA-23/patA-24 (for *patA*). Both fragments were cloned into plasmid pCSEL22 (28), rendering plasmid pCSAV281, which contains a fusion of the *ftsZ* promoter region and sequences encoding the first six amino acids of FtsZ and promoter-less *gfp*-*mut2*, and plasmid pCSAV280, which contains a fusion of the *patA* promoter and sequences encoding the first six amino acids of PatA and promoter-less *gfp-mut2*. All plasmids used in this work were verified by sequencing. Plasmids pCSAV281 and pCSAV280 were transferred by conjugation (29) to *Anabaena* sp. strain PCC 7120, with selection for Sm and Sp (resistance is encoded in the portion vector of the transferred plasmids), and thus for clones that had the transferred plasmid inserted into the homologous genomic region by a single-crossover event. One clone that included the P*_ftsZ_-gfp-mut2* construct and one that included the P*_patA_-gfp-mut2* construct were selected and named strains CSAV43 and CSAV45, respectively.

**TABLE 1:** Oligodeoxynucleotide primers used in this work

Primer name	Primer sequence (5′–3′)^*a*^
patA-7	GCGATCGCCTGCAGAACACTTCCG
patA-8	GTAATAGTTGAGAATTCGTAATGTG
patA-9	GCGATCGCTGCAGAAACACTTCCG
patA-10	GTGAATTCTTATTACGTAATGTG
patA-11	AAGCAAGGTCTCACGCCCGTAATGTGTTTAAA
patA-14	ATTATAAAGCGGCCGCCATTAACTTGGACTTTSCCC
patA-17	GCCATGAAAACACTTCCGATTAC
patA-18	ATCGCTGCAGTTACGTAATGTGTTTAAA
patA-19	AACGGGATCCGGCGATCGCTCTGTTATT
patA-20	TTCGCTGCAGCTTTCAGGTTAATCTTTTv
patA-21	GCAACATATGAAAACACTTCCGATT
patA-22	CGTTGGATCCTTATTACGTAATGTGTTTAAAAAT
patA-23	ATGGATATCAATCGGAAGTGTTTTCAT
patA-24	GATATCGATGGTTTCTCCTGTACGGTTT
sfgfp-12	TTCGGGATCCATGGCTAGCAAAGGAGAAGAA
sfgfp-13	GCCTCCACCGCCTTTGTAGAGCTCATC
sfgfp-14	GCACTGCAGGGCCTCCACCGCCTTTGTAGAGCTC
sfgfp-15	ATCATGAGCAAAGGAGAAGAA
ftsZ-52	GTAATCGATGTAGTACGTTTCCAGTGGC
ftsZ-53	ATGGATATCGTTATTATCAAGTGTCAT
zipN-24	GCAACATATGTTGATCACGGTGCAG
zipN-25	CGTTGGATCCTTATTAATTTATAGCGGCTGA
zipN-26	GAAGGTACCGCCCCAAAGTCATGTCTTCG
zipN-27	ATCAATTCACCTAGACCATTCCAG

a Underlined letters indicate a restriction site.

For generation of an *Anabaena* derivative expressing a fusion of the FtsZ protein to GFP, plasmid pCSSC39 (containing the *gfp-mut2* gene, preceded by four Gly-encoding codons, fused to the last codon of the *Anabaena ftsZ* sequence) (19) was transferred to strain UHM101 (*patA* mutant [21]) with selection for Sm and Sp, generating strain CSAV38.

For generation of an *Anabaena* strain expressing a fusion of the ZipN protein to sfGFP, a DNA fragment containing the *zipN* promoter sequence and another containing the *sf-gfp* gene were amplified by PCR using *Anabaena* genomic DNA as the template and primers zipN-26/zipN-27 or using plasmid pCSAL39 (which includes the sfGFP-encoding sequence preceded four Gly-encoding codons [13]) and primers sfgfp-14/sfgfp-15, respectively. These fragments were joined together by the use of overlapping PCR with primers sfgfp-14/zipN-26 and cloned into pSpark vector, from which the construct was excised, with PstI ends, and transferred to PstI-digested pCSS192 (which contained the *zipN* open reading frame [ORF] cloned into vector pKT25 [20]), producing plasmid pCSAV284. Finally, the KpnI-ended P*_zipN_*-*sf-gfp-zipN* construct was transferred to conjugative plasmid pCSV3 (30), producing pCSAV285, which was transferred to *Anabaena* by conjugation with selection for Sm and Sp, generating strain CSAV39.

Two *Anabaena* strains expressing fusions of the PatA protein to sfGFP were generated. For the *patA-sf-gfp* construct, a DNA fragment was amplified using *Anabaena* DNA as the template and primers patA-14/patA-11 and was cloned in plasmid pCSAL39. The KpnI-ended P*_patA_-patA*-*sf-gfp* construct was transferred to pCSV3, producing pCSAV255, which was transferred to *Anabaena* by conjugation with selection for Sm and Sp, generating strain CSAV35. For the *sf-gfp-patA* construct, a DNA fragment containing the *patA* gene and another containing the *sf-gfp* gene were amplified by PCR using pCSAV255 as the template and primers patA-17/patA-18 (for *patA*) or plasmid pCSAL39 and primers sfgfp-12/sfgfp-13 (for *sf-gfp*). These fragments were joined together by overlapping PCR with primers sfgfp-12/patA-18 and cloned into pSpark vector, rendering plasmid pCSAV272. A third DNA fragment (which included the *patA* gene promoter) was amplified using *Anabaena* DNA and primers patA-19/patA-20 and cloned in BamHI-digested pCSAV272. Finally, the P*_patA_*-*sf-gfp-patA* construct was transferred to conjugative vector pRL277 (31), producing pCSAV271, which was transferred to *Anabaena* by conjugation with selection for Sm and Sp, generating strain CSAV41.

### BACTH assays.

BACTH assays based on the reconstitution of adenylate cyclase from Bordetella pertussis (32) were performed with genes amplified by PCR and cloned in pUT18, pUT18C, pKNT25, or pKT25, producing fusions to the 5′ end or 3′ end of the adenylate cyclase T18 and T25 fragments. Primer pairs used for amplification of *patA* were as follows: patA-7/patA-8 (T18-PatA, PatA-T18, and PatA-T25) and patA-9/patA-10 (T25-PatA). The resulting plasmids were verified by sequencing and transformed into E. coli XL-1-Blue for amplification. Fusions to FtsZ, ZipN, SepF, and Ftn6 (20) and to SepJ (33) were previously described; fusions to FraC and FraD (J. E. Frías and E. Flores, unpublished data) are to be described elsewhere. Isolated plasmids were cotransformed into strain BTH101 (*cya-99*), and the transformants were plated on solid LB medium containing selective antibiotics and 1% glucose. β-Galactosidase activity was measured in liquid cultures incubated at 30°C in the presence of IPTG (isopropyl-β-d-thiogalactopyranoside) and antibiotics, using *o*-nitrophenyl–β-galactoside as the substrate (33). The amount of *o*-nitrophenol (ONP) produced per mg of protein versus time was represented, and β-galactosidase activity was deduced from the slope of the linear function.

### Copurification assays.

For expression of *Anabaena* ZipN N-terminally fused to 6×His, the *zipN* gene was amplified by PCR using plasmid pCSS192 and primers zipN-24/zipN-25 and cloned in pET28b (Novagen), producing plasmid pCSAV277. For expression of a version of *Anabaena* PatA N-terminally fused to Strep-tag II, the gene *patA* was amplified by PCR using plasmid pCSAV255 as the template and primers patA-21/patA-22 and cloned in the pCMN28b expression vector, which is based on pET28b and carries the Strep-tag II-encoding sequence instead of 6×His-encoding sequence (34), producing plasmid pCSAV276. Both pCSAV276 and pCSAV277 were transformed into E. coli BL21.

For preparation of cell extracts, E. coli cells bearing pCSAV276 or pCSAV277 were incubated for 20 h at 24°C after the addition of 1 mM IPTG. Then, cells were harvested by centrifugation for 10 min at 5,000 rpm. The cell pellets were washed with buffer A (50 mM Tris-HCl [pH 7.5], 150 mM KCl, 10% glycerol) and resuspended in ice-cold buffer A (5 ml g^−1^ of cells) containing protease inhibitor cocktail complete Mini EDTA-free (Roche) and homogenized on ice for 5 min. The cell suspensions were incubated with 1 mg ml^−1^ lysozyme for 1 h at 4°C and were subjected to six pulses, 30 s each, of ultrasonication. The lysates were cleared by centrifugation at 15,000 × *g* for 30 min at 4°C. For copurification assays, aliquots of the resulting cell extracts were mixed and incubated overnight at 4°C. As a control, each cell extract alone, or an extract from E. coli (including pCSAV277) mixed with an extract from E. coli (including pET28b plasmid vector), was incubated overnight at 4°C. Then, extracts were passed through a 1-ml His-Select column (Sigma) following the instructions of the manufacturer, and the material retained was washed and eluted with buffer A supplemented with increasing concentrations of imidazole. Aliquots of the eluted fractions were resolved in 10% SDS-PAGE gels and subjected to Western blotting with a commercial antibody against Strep-tag (Qiagen).

### Microscopy.

Cell area data were estimated automatically with ImageJ (https://imagej.nih.gov/ij/index.html) processing of light-microscopy images (20), and data were plotted using RStudio Desktop software. GFP fluorescence and *Anabaena* red autofluorescence were visualized with a Leica DM6000B fluorescence microscope equipped with a fluorescein isothiocyanate (FITC) L5 filter (excitation, 480/40 nm; emission, 527/30 nm) for GFP and a Tx2 filter (excitation, 560/40 nm; emission, 645/75 nm) for autofluorescence, and the results were photographed with an Orca-ER camera (Hamamatsu). Alternatively, fluorescence was monitored with a Leica TCS SP2 confocal laser scanning microscope equipped with an HCX PLAN-APO 63× 1.4-numerical aperture (NA) oil immersion lens objective. Samples were excited with 488-nm irradiation from an argon ion laser, and fluorescence was collected across windows of 500 to 540 nm (GFP) and 630 to 700 nm (cyanobacterial autofluorescence). For quantification of GFP fluorescence, ImageJ software was used to compile fluorescence in manually defined areas along filaments.
